# The Impact of Relational Coordination on Staff Outcomes in Long-Term Care

**DOI:** 10.1093/geroni/igaf122.4390

**Published:** 2025-12-31

**Authors:** Lisa Cranley, Daniyal Zuberi

**Affiliations:** University of Toronto, Toronto, Ontario, Canada; University of Toronto, Toronto, Ontario, Canada

## Abstract

Relational coordination (RC) emphasizes the importance of communication and relationships among staff to effectively coordinate care. This study investigated the impact of RC on staff outcomes: work engagement, job satisfaction, and burnout in seven long-term care (LTC) homes in Ontario, Canada. In phase 1 of this mixed-methods study, an online survey was distributed to staff (nurses, allied health providers, personal support workers) working in LTC homes. Staff were asked about RC, work engagement, job satisfaction, work-related burnout (professional efficacy, emotional exhaustion, cynicism), and demographic information. The impact of RC on staff outcomes was assessed using random-effect modeling to account for the multi-level (staff/ LTC) data, with staff as the unit of analysis and LTC as the random effect. We received 153 completed surveys. RC had a significant positive association with staff work engagement, job satisfaction, and professional efficacy in both unadjusted and adjusted models for staff demographics and LTC size. In contrast, RC had a significant negative association with emotional exhaustion and a non-significant negative association with cynicism. Age had a significant positive association with work engagement and job satisfaction, while the association was inverse with cynicism. Holding a bachelor’s degree or higher had a significant positive association with work engagement and job satisfaction; however, the association was inverse with emotional exhaustion. None of the demographics were significantly associated with professional efficacy. Better communication and relationships between staff can enhance their work engagement and well-being, which could improve overall work efficiency and support quality improvement innovations for integrated care.

